# Ethical Issues in Patient Data Ownership

**DOI:** 10.2196/22269

**Published:** 2021-05-21

**Authors:** Varsha Chiruvella, Achuta Kumar Guddati

**Affiliations:** 1 Augusta University Augusta, GA United States

**Keywords:** data, privacy, ownership

## Abstract

Patient data have conventionally been thought to be well protected by the privacy laws outlined in the United States. The increasing interest of for-profit companies in acquiring the databases of large health care systems poses new challenges to the protection of patients’ privacy. It also raises ethical concerns of sharing patient data with entities that may exploit it for commercial interests and even target vulnerable populations. Recognizing that every breach in the confidentiality of large databases exposes millions of patients to the potential of being exploited is important in framing new rules for governing the sharing of patient data. Similarly, the ethical aspects of data voluntarily and altruistically provided by patients for research, which may be exploited for commercial interests due to patient data sharing between health care entities and third-party companies, need to be addressed. The rise of technologies such as artificial intelligence and the availability of personal data gleaned by data vendor companies place American patients at risk of being exploited both intentionally and inadvertently because of the sharing of their data by their health care provider institutions and third-party entities.

## Introduction

The history of patient records dates back 4000 years, when patient case records were stored in written form [1]. Unlike the modern technologically driven age, in ancient times, caregivers relied heavily on paper-derived means to maintain patient records. For example, ancient Egyptian hieroglyphics from 1600 to 3000 BC indicated that patient reports were inscribed on papyri [2]. In America, the clinical record pioneered major teaching hospitals in the 19th century, whereas medical records for direct patient care later developed in the 20th century. At this time, health records were traditionally written on paper, tediously organized, and divided into folders with only one copy per note. In an increasingly technological era, beginning in the late 20th century and the beginning of the 21st century, problems with the way patient records were documented began to emerge. Illegible handwriting and the inability to easily share, permanently store, and retrieve necessary information were some challenges faced in the predigital era. Studies from the time before technological development stated that tests were often reordered because of missing, illegible, or inaccessible components in patient records. One report from the late 20th century noted that 11% of laboratory tests were duplicated in one hospital because of unavailable information for the physician [3]. These were some of the driving factors in the need for a better health care system.

The trend toward automation of patient data recording coincided with the appearance of multiple new forms of reporting. Computers were introduced into hospital settings and used for administrative and financial purposes in the 1960s, with the goal of reducing clerical error and improving clinical decision making. The introduction of the electronic health record (EHR), and its less comprehensive counterpart electronic medical record (EMR), in the United States in the 1970s revolutionized the way patients were documented and treated. Although frequently interchanged, EMR refers to a digital record of a patient’s treatment at a specific institution, whereas electronic health record is a complete longitudinal record of a patient’s medical history and treatment. The Institute of Medicine (which changed its name to National Academy of Medicine in 2015) reported that information technology is essential for quality patient care. The dawn of a digital age provided medical professionals an opportunity not only to obtain a greater depth of medical knowledge but also to access patient information almost effortlessly. With such technological features, physicians can now easily acquire a patient’s list of allergies, medications and dosages, and past medical and surgical histories. EMR changed how the medical world maintains patient records by establishing an ease and convenience in how health reports are read and accessed today. However, there is debate in the United States on whether EMR is beneficial for patients [4]. In particular, there are arguments that digitalization may come at the price of patient privacy. A balance between upholding patient privacy, autonomy, and furthering medical knowledge through research and providing efficient, beneficial patient care, as outlined in the principle of beneficence, has become an increasingly important topic because of the rise of advanced technology integration into medical practice.

## Legal Considerations for Patient Data in the United States

What distinguishes patient data, in particular, from browsing data and metadata is the legal binding of patient-physician confidentiality because of the provisions in the 1957 Code of Medical Ethics of the American Medical Association, section 4. In 1996, the Health Insurance Portability and Accountability Act (HIPAA) was established. The HIPAA Privacy Rule protects all “individually identifiable health information” and “protected health information” (PHI) held or transmitted by a covered entity or its business associate, in any form or media, whether electronic, paper, or oral. The Privacy Rule provides national standards and safeguards to protect individuals’ personal health information and medical records. It sets the limits and conditions that govern the appropriate disclosure of such information with and without patient authorization. In addition, although HIPAA does not regulate the retention of information, there are legal requirements in place under the Code of Federal Regulations (CFR), namely 45 CFR §164.316(b)(1), for holding specific patient data in technology for a certain period. Thus, there exists a certain life cycle for patient records that consists of creation, utilization, maintenance, and ultimately destruction. This step-by-step management protocol is implemented through health record retention plans to make health information retrieval efficient and rapid. Plans address what data should be available that meet the required functions, such as continued patient care and legal purposes, time frames for data maintenance and destruction, and data destruction policies and procedures. Retention plans, such as the template of the Higher Education Act of 1965, must meet federal record retention requirements, state record retention requirements, and many disclosure requirements [5]. Legal requirements for holding patient data depend on federal and state requirements, which are specific to the type of documentation. For example, although records of patients with end-stage renal disease services must be maintained for 6 years, data on hospital radiologic services such as films and scans are maintained for 5 years [5]. Once a document has met its full retention period, an organization must ensure that paper and electronic records are destroyed in accordance with federal and state laws. Some methods of destruction include shredding and burning of paper records, pulverizing microfilm and laser disks, and magnetic degaussing of computerized data. However, anonymization is not currently considered a form of destruction.

The government also regulates the process of sharing patient information. Essentially, the Privacy Rule controls who can view and receive a patient’s health information, including electronic, written, and oral forms. However, this rule presents additional problems with regard to confidentiality concerns. For example, there is an underlying challenge of protecting patients’ privacy while communication occurs among health care providers, insurers, policyholders, and patients. Sharing confidential and sensitive patient information could affect patient coverage, billing, and claims processes. There is a possibility that disclosure results in denied justice, equity, or fairness based on shared sensitive patient data such as on sexual and reproductive health, mental health services, and substance abuse treatment. Although health care providers normally seek patients’ consent when disclosing patient data for health insurance claims, the HIPAA Privacy Rule allows disclosure of PHI without patient authorization with organizations subject to the Privacy Rule, termed *covered entities*, for operations of treatment, payment, public safety, or requirement by law [6]. It should be noted that under 45 CFR §164.514, patient data in a HIPAA-limited data set can be shared without consent, and covered entities under the Privacy Rule include physician offices, clinics, psychologists, insurance companies, nursing homes, health care clearinghouses, and government agencies that contribute to health care. In all other cases, patient consent is required for the disclosure of information. An example of the exception under HIPAA for patient authorization is the requirement of insurers to send policyholders’ explanation of benefits, which details service billing. As a result, the required disclosure of patient-sensitive information may have an effect of deterring or denying health care coverage. In addition, sharing sensitive patient information outside of the scope of the provider and patient runs the risk of stigmatization and discrimination in vulnerable populations and law enforcement involvement, such as in cases of immigration status. Physicians in cases such as these must balance the professional and ethical responsibilities of justice to provide quality care to all people regardless of their background [7].

## Examples of Current Data Utility: Sharing and Distribution of Patient Data

Collecting patient data is fundamental in health care to provide the best and most appropriate care. It must also be conducted appropriately in a HIPAA-compliant manner. There are several software tools and research networks in the United States, such as Research Electronic Data Capture (REDCap), Research Action for Health Networks (REACHnet), and Agency for Healthcare Research and Quality (AHRQ), which demonstrate how patient data may be shared responsibly. Pooled patient data can help providers and researchers better recognize health issues, identify symptom similarities, advance treatment options, conduct studies, report trends, and stay updated with the current literature, with the hope of improving patient outcomes. This is the basis for software programs such as REDCap. REDCap is a secure and intuitive web app created by Vanderbilt University for capturing data for clinical research and building and managing web-based surveys, databases, and projects. Most importantly, it is a highly secure data collection tool that complies with HIPAA and supports single- and multiple-site research studies. REDCap allows all project data to be stored at a local institution, while no data are transmitted from that institution to third-party institutions or organizations. Thus, it is limited to intrainstitutional study. In addition, patient information in REDCap can be marked as identifiable but can be easily deidentified by the user during export, providing safe intrainstitutional privacy and security [8]. Unlike the intrainstitutional limitation of REDCap, REACHnet is an interinstitutional data network consisting of multiple health systems, academic centers, and health organizations. Similar to REDCap, REACHnet’s function is to conduct efficient yet multisite research to implement more effective health care decision making and improve population health.

Furthermore, the AHRQ has a mission to improve health care quality and make care more accessible, affordable, and equitable. The agency invests in health systems research and analyzes data to aid health care decision making and creates strategies to improve medical practice. The Healthcare Cost and Utilization Project (HCUP) is a collection of databases sponsored by the AHRQ. The HCUP network contains both clinical and nonclinical patient details, including patient demographics, diagnoses, procedures, charges, and insurance information. Thus, the HCUP enables research that focuses on many current health care policy issues such as access, cost, and quality of care [9]. An example is a statistical brief published in 2017 by the HCUP, which discusses the costs of emergency department (ED) visits for those with mental and substance use disorders. It reports that the rate of ED visits for mental health and substance abuse diagnoses increased by 44.1% from 2006 to 2014, translating to 20.3 visits per 1000 individuals [10]. Such studies by the AHRQ and its partner, the US Department of Health and Human Services, are focused on health policy concerns to improve, for example, ED service delivery costs for patients. In light of its beneficial nature in aiding policy decisions, patient data must be collected from the AHRQ databases. However, this can result in high costs. Depending on the scope of the reports, the cost of obtaining simple or comprehensive reports can vary, ranging from thousands to hundreds of thousands of dollars [11]. AHRQ, in addition to REDCap and REACHnet, presents a compelling argument on the benefit of patient data utility for health care improvement as well as examples of appropriate HIPPA-compliant use of patient information. These examples are in contrast with patient privacy problems found within the overlap of big tech companies and health care.

## The Intersection of Big Tech and Health Care: Implications and Complications

The topic of patient privacy in technologically available patient data has gained traction in recent years, given the recent advances in big tech industries in health care [12]. The sale of patient data to commercial companies, such as Amazon, by hospitals and hospital networks has many disadvantageous implications. First, patient data may be exploited with unauthorized access by third parties (hackers). Second, individuals may lose control over their data when data collection companies are purchased by other companies. In these cases, the purchasing company gains access to the patient data and can use these data without the consent of the individuals in question. Third, there is a possibility that data that were anonymized and deidentified by these companies are reidentified. Data breaches in patient data may also result in the targeting of vulnerable populations and discrimination.

Big tech companies such as Amazon claim to enter the health care field for the benefit of the medical system, which is currently unable to synthesize the enormous patient database that is available. Another benefit of the technology industry is the ability to use medical data to develop new drugs, devices, and algorithms to help diagnose disease and help future patients. In particular, *Amazon Comprehend Medical* is Amazon Web Service developed to assist the medical system overwhelmed by patient information. The goal of Amazon Comprehend Medical is essentially to organize patient information into customized databases specific for pharmaceutical companies, hospitals, and researchers. Amazon’s cloud service with advanced machine learning can theoretically *read* uploaded patient documents, identify the type of data, and categorize it into a database. This advanced program can pull key data points from unstructured health care data and published research. Comprehend Medical also helps the customer or, in this case, the patient. The service provides a platform for patients to easily gather information on their medical condition and appropriate medication and dosages from Amazon’s database of doctor notes, clinical trial reports, and health records.

With Amazon’s global reach and widespread user network, it is concerning how the company may not be doing enough to protect patient privacy and may misuse information for advertising purposes. Amazon claims that Comprehend Medical is HIPAA-eligible and can easily identify PHI before patient information is stored. As stated in HIPAA, PHI is based on a list of 18 identifiers (ie, name, age, and relevant dates) that can be used to recognize the identity of a patient and must be treated with special attention. Although Amazon reports that these identifiers can be detected, entities may not always map accurately to the list specified by Amazon’s DetectPHI operation. In other words, Amazon Comprehend Medical contains all the relevant identifiers, but not all identifiers may be recognized and removed [13]. Furthermore, to protect patient privacy, federal restrictions are in place to prevent the use of medical data for marketing or any commercial purpose beyond patient care. Amazon reports that the cloud over which patient data are transferred does not collect or store any data processed by Comprehend Medical. However, it may be difficult to believe that PHI would not be used for product or service marketing given Amazon’s heavy presence in the commercial world. One’s apprehension toward Amazon Comprehend Medical may be furthered by the fact that it is *HIPAA-eligible* rather than HIPAA-compliant. HIPAA-eligible means that it is the responsibility of the customer, medical institution, or health care organization that sells data to Amazon to ensure that it complies with patient privacy regulations [14]. Amazon may not be able to fully deidentify protected patient information; therefore, it is essentially on the patient and the providers to ensure compliance and uphold patient privacy.

As technology companies are increasingly merging with the health care field, concerns over patient privacy have become increasingly valid. First, patient information may be misused once personal data are shared in corporate mergers. In addition, patient data may be misused because of fraud and unauthorized access. For example, Amazon purchased the web-based pharmacy company PillPack in 2018 for approximately US $750 million, thereby inserting Amazon’s dominance into supply chain management and delivery services. As a result of the acquisition, patient data and insurance information went to Amazon rather than a pharmaceutical company. PillPack functioned in combination with the third-party intermediary ReMyHealth and SureScripts, a company that gathered patient medical documentation and web-based prescriptions. PillPack used ReMyHealth to obtain patient data collected by SureScripts until 2019, when it was discovered that ReMyHealth had been involved in fraudulent activities. SureScripts alleged that ReMyHealth had provided unauthorized access to patient health information and exploited prescription information for marketing purposes. An investigation into ReMyHealth revealed that the company’s fraud had manifested as several thousand requests for patient health insurance information and prescription drug price information, which was provided by ReMyHealth to other parties for marketing specific medications to consumers. Consequently, SureScripts terminated its contract with ReMyHealth. As ReMyHealth was the third-party company responsible for PillPack’s information about patient prescriptions, SureScripts’ termination with the company resulted in a blow to Amazon’s PillPack, as it no longer had a clear or efficient way to access data [15].

Another intriguing example of a company accused of sharing and selling patient information, fraudulently or through deals with pharmaceutical companies, is 23andMe, a popular personal genomics and biotechnology company [16,17]. Companies such as 23andMe have made the discovery of an individual’s ancestry as easy as swabbing one’s cheek or spitting in a cup. However, similar to PillPack, ancestry discovery sites are not immune to fraud and confidentiality breaches. In fact, there was a privacy breach in 2017, in which more than 92 million accounts from the DNA testing service MyHeritage were found on a private server [18].

In addition to aiding in personal discovery, the company provides consumers the choice to opt for research conducted on behalf of academic and nonprofit organizations. It is no secret that DNA testing companies such as 23andMe and Ancestry share anonymized consumer genetic information with pharmaceutical giants such as GlaxoSmithKline, companies such as P&G Beauty, and university and research institutions as part of million-dollar deals, resulting in a further reduction in patient awareness and control of their own data utilization [19]. A main reason why consumers may choose to participate in research opportunities and discovery is simply consumer altruism toward improving health care and scientific knowledge. One may believe that if their DNA could help find the cause of, or a cure for, a disease, it would be worthwhile to contribute their genetic information. However, when a drug company actually brings a drug to market based, in part, on one’s DNA, the general population will not be afforded a cheaper medication despite their altruistic efforts. Thus, in addition to the possibility of inappropriate distribution and commercial use of secure patient data, the fact that patients receive no financial compensation for the use of their own data provides a depth of complexity to the sharing and utilization of electronic health information. Simply put, patient privacy concerns may conflict with the advancement of knowledge through data sharing.

To further complicate matters on confidentiality, HIPAA’s provisions for data protection do not necessarily mean that data are anonymous. For instance, deidentified patient data on Amazon Comprehend Medical may not remain anonymous. HIPAA-eligible Comprehend Medical can identify and redact certain PHIs to make web-based patient data anonymous. However, it is possible to reidentify patients from deidentified data [20-22]. A 2000 study from Carnegie Mellon University showed how anonymized US census data could identify some individuals simply by combining a few demographic details, such as city of birth and zip code [21]. Researchers in Europe have also claimed that they were able to correctly identify 99.98% of Americans in deidentified data sets using 15 demographic attributes [22].

## Commercial Targeting of Vulnerable Populations: A Risky Possibility

Sharing sensitive patient information with other agencies and organizations could put vulnerable populations at risk. For example, agencies could potentially monitor sensitive demographic information such as transgender status and immigration for nonhealth purposes. The possibility of pharmaceutical companies using patient information to target vulnerable populations is also a relevant concern. In particular, in vulnerable populations, sensitive and confidential patient data may be used to deny justice, equality, or fairness. However, what is a vulnerable population? The term implies a disadvantaged subpopulation that requires more care, consideration, and protection in health care because of the risks of poorer health status, health care access, and life expectancy [23]. Older people, pregnant women, children, prisoners, minorities and refugees, and those with chronic illnesses are some examples of vulnerable populations. Sensitive information that vulnerable patients may fear of being monitored or exploited include history of domestic violence or substance use, genetic information, mental health information, sexual orientation, and immigration status. In addition to facing inequalities and provider bias, vulnerable populations might also have concerns regarding the use of patient data for profit utilization.

A contemporary example of health care systems targeting a vulnerable population for commercial purposes is that of recovering alcoholics and those with a history of substance use disorder. Alcohol or cigarette companies can exploit this addiction after disclosing individuals’ past medical history by providing triggering advertisements and marketing their products. If patient health care information is integrated into a technological world driven by business, it may not be difficult for pharmaceuticals to exploit sensitive information, such as sexuality, transgender status, immigration status, and history of substance use, and nonsensitive patient information, such as age and gender. One real case illustrating this possibility is that of Avanir Pharmaceuticals. In 2019, the company was charged with paying physicians kickbacks to promote prescriptions of its drug Nuedexta, primarily targeting long-term care facilities with older patients who may have presented with signs of dementia. However, the drug had no proven use in dementia treatment, and its purpose was clouded by the company’s false and misleading information [24]. The purpose of these kickbacks was to raise Avanir Pharmaceutical’s sales at the expense of the vulnerable older people and nursing care population. Furthermore, the state of Pennsylvania sued the pharmaceutical company Purdue Pharma in 2019 over claims that the company mass produces the drug OxyContin, thereby fueling the state’s deadly opioid epidemic. Purdue allegedly targeted physicians and focused on the geriatric and veteran populations, assuring them that the drug was not addictive and downplaying any risk [25]. These are a few examples that indicate the risk associated with the commercial sharing of patient information that may be exploited by third-party organizations such as pharmaceuticals for commercial purposes. Vulnerable populations, such as older people, are more at risk than the average individual of being harmed by unethical marketing through manipulation or deception. Despite guidelines and legal requirements in place to protect vulnerable populations in fields such as labor and research (ie, Genetic Information Nondiscrimination Act, Americans with Disabilities Act, and Patient Protection and Affordable Care Act), vulnerable populations’ health data are not protected on the web under these provisions.

It is important to note that pharmaceutical companies profiting from patient information do not necessarily need a comprehensive medical history or access to sensitive patient information to commercially target populations. Rather, drug companies can use browsing history, age, gender, and locations to piece together an individual’s health issues and market appropriately. The power of advertising on pharmaceutical wealth has also been studied. For instance, a study by the Wharton school and the University of Southern California estimated that for every 10% increase in advertisement exposure, there was a corresponding 5% increase in the number of prescriptions purchased [26]. What lies behind what one sees on their computer screen is around a billion dollars spent by pharmaceutical companies and health care brands every year to market their goods on Facebook [27]. Although the pharmaceutical industry spent US $59 million on direct-to-consumer advertising on the internet in 2003, this number has risen to US $1 billion in recent years [28]. It is possible that direct-to-consumer drug advertising efforts on the internet have expanded, as searching for health-related information may become an increasingly common activity for web-based users. This presents an ethical *gray area* in terms of patient data and privacy, as even HIPAA does not address the crossing of drug companies and social media outlets. In addition, Amazon and companies such as Google and Microsoft have also purchased access to patient data. Just as social media platforms and pharmaceutical companies can exploit patient browsing history, tech companies such as these may pose similar privacy risks through the sharing of patient health information.

## Potential for Improvement of Health Care Quality

Despite concerns about patient privacy, the integration of technology and medicine could improve the quality of health care. EMR and the benefit of Amazon Comprehend Medical in restructuring its data on both the patient and provider ends could empower a consumer to take charge of their own well-being and be more proactive in maintaining their health. Although it comes at a price of privacy, sharing patient information could equip consumer patients and partner organizations with more information about their health with the help of artificial intelligence (AI). Even nonhealth care–related data, such as patient habits and search history, could provide useful information. For instance, health care organizations could market cold and flu medicine to someone who frequently books appointments at the beginning of the flu season or recommend obstetricians to someone who recently bought prenatal supplements and pregnancy tests [29]. This type of predictive technology through AI can be used to help prevent hospital readmissions and identify at-risk patients. AI technology in health care could also enable the discovery of new patterns of disease, pathogenesis, and treatment. Some key categories of AI applications involve diagnosis and treatment recommendations, patient engagement and adherence, and administrative activities [30]. One of the most popular and increasingly relevant forms of AI in health care is machine learning and its application in precision medicine. Precision medicine in health care allows for the prediction of treatment protocol success based on patient traits and the context of the treatment. IBM Watson has gained much attention in the media because of its capability of precision medicine for cancer diagnosis and treatment. Google is also deriving an AI algorithm to create a prediction model that can alert physicians of high-risk conditions such as sepsis and heart failure [30]. Despite such immense achievements, full integration into health care processes and systems remains a challenge. Furthermore, although AI may have a future role in enabling the discovery of new disease patterns, pathogenesis, and treatment regimens, privacy and confidentiality risks remain ethical concerns in the field of AI in health care.

## Privacy Solutions

The topic of patient privacy, in conjunction with the rising use of electronic records and the increasing realm of big tech companies, highlights a relevant point of study on whether sharing health care information does more harm than good. Patients generally want to share data to improve health care but want more control over sharing their personal health information. Thus, sharing clinical data should involve a degree of transparency in patient compliance. One study reported how respondents felt comfortable participating in research if they provided information about what aspects of their data were being shared and with whom. The respondents were healthy volunteers who had responded to posted advertisements around the University of California San Diego within 4 months. A total of 83% showed a strong preference for the control of specific data, whereas 68% were concerned about the possibility of their information being used for commercial purposes [31]. Another study reports how patients prefer sharing their information with *granular* privacy control over which data would be shared and with whom. In addition, individuals have differences in preferences for which type of EMR data is shared. Regardless of whether individuals had sensitive information on record, they were less likely to want to share sensitive information when compared with nonsensitive information [32]. A study by Whiddett et al [33] supports this finding with a 2016 study of 4209 adults in New Zealand. This survey revealed that individuals are significantly more likely to share their data with nurses, doctors, and paramedics than with government agencies. In addition, individuals with sensitive information on records were significantly less likely to consent to sharing their records [33]. A large proportion of the population, especially vulnerable populations, is reluctant to share their records beyond health care professionals. Widespread distribution of patient information across platforms such as billing and insurance purposes, pharmaceutical involvement, and big tech companies may thus have adverse effects on the levels of patient trust in health care as well as the equal and fair treatment of patients in other facets.

It would be in patients’ best interests to be actively involved in the development of policies on data sharing. Improving patient awareness about the type of data and nature of information contained in their records would be an appropriate measure, in addition to information regarding to whom their records are sent. Researchers should ensure that patients are given adequate informed consent regarding which aspects of their information are being used when seeking consent for data extraction. Maintenance of transparency among patients, providers, and research institutions is important. Patients should not only be notified when their data are used in research but also informed of the outcomes and future implications of this research. This, by definition, encompasses the solution of *dynamic* consent or the approach to informed consent that enables streamlined, continuous involvement and communication between individuals and the users of their data [34,35].

However, the transparency of dynamic consent is complicated by several factors such as the biases that individuals hold in sharing information; the question of what qualifies as *adequate* informed consent, including addressing various educational competencies; and differing expectations that individuals may have toward providing consent, which may involve varying expectations of their freedom to change the levels of consent or engagement [36,37]. Furthermore, big tech companies may attempt to share the least amount of information possible with individuals who still comply with consent requirements. First, the results of surveys that reveal how individuals are more likely to share their data with health care professionals may undermine or call into question the reliability and effectiveness of the obtained consent. Second, it is difficult to quantify or measure the extent of *adequate* consent. For instance, users of Amazon Comprehend Medical may not be aware of or understand the difference between HIPAA-compliant and HIPAA-eligible before providing the big tech company their sensitive health information. In this case, it is the company’s duty and responsibility to inform all users of the risks in sharing patient information with the company and define their terms of HIPAA eligibility. Finally, individuals may have varying expectations on what is to be informed of them regarding the utilization and distribution of patient data. Although some may provide consent to many uses of their data with minimal disclosure, others may adopt a more limited approach to consent, with expectations of full transparency on use and anticipation of potential financial compensation before consenting. These differences in consent within a population make reevaluation of consent requirements more challenging. Thus, informed consent is difficult to generalize to a population. A solution to this dilemma is the model of *meta consent* as part of a smartphone app. The idea behind meta consent is that individuals should be asked how and when they would like to provide consent. It allows the patient to choose from a list of types of consent (specific consent, broad consent, blanket consent, and blanket refusal in the context) in the context of electronic patient records, data from samples, and commercial research. The meta consent app, a model successfully tested in an adult Danish population, is sensitive to individual consent preferences and caters to a wide variety of expectations regarding consent. Meta consent also allows greater transparency between individuals and data holders and places more control in the hands of individuals in choosing the terms of data use [38]. In addition to the meta consent model, individuals should be given the right to access, amend, and delete individually identifiable data held by data custodian or third-party processors. As such, this model should be used to collect consent preferences in the US population.

Given that HIPAA has not effectively protected patient information in several aspects such as vulnerable populations and in the realm of big tech, social media, and pharmaceutical involvement in health care, HIPAA laws should be amended to reflect current times. First, the definition of personal health information should be expanded to include broader protection for individuals. In this model, HIPAA would be revised to more closely resemble the 2018 California Consumer Privacy Act or the European Union’s General Data Protection Regulation of *data concerning health* rather than the traditional American protection of data limited to health care [39,40]. This means that the 1996 law should add provisions detailing protection from the intersection of health-related Google searches and personal spending and commercial targeting. Essentially, web searches on a rare disorder and insurance coverage or buying a box of pregnancy tests should not result in increased web-based advertising of baby products or pharmaceutical endorsements. In addition, owing to the elevated concerns and apprehension of individuals toward sharing data with highly sensitive information, HIPAA should do more to protect vulnerable populations. Extra provisions should protect sensitive information from solicited distribution, such as between covered entities outlined in the Privacy Rule, and unsolicited distribution, such as data breaches and unauthorized sharing, of patient information that could result in altered insurance costs or any other form of inequality or unjust treatment.

## Conclusions

This paper revealed the underlying conflict between what is overwhelmingly considered ethical in health care: patient autonomy and right to privacy, or beneficence, the ethical responsibility to do more good than harm. The integration of big tech companies such as Amazon into the realm of health care has many implications on confidentiality but could also have potential for advantageous discovery. We believe that collaboration on patient information on different fronts, such as the technological industry and medical centers, can provide valuable information that can enhance knowledge through research and improve patient-based care. However, digitalization and sharing of patient information have privacy implications that need to be addressed and fixed with modified provisions under HIPAA as well as enforcement of informed consent with flexibility in patient preferences. There are many factors that need to be considered legally and socially in terms of patient relationships when health information is shared with third parties, whether big tech, pharmaceuticals, or insurance companies. The rise of advanced technology in the 21st century presents this discussion as more relevant than ever.

## Abbreviations

AHRQ: Agency for Healthcare Research and Quality
AI: artificial intelligence
CFR: Code of Federal Regulations
ED: emergency department
EMR: electronic medical record
HCUP: Healthcare Cost and Utilization Project
HIPAA: Health Insurance Portability and Accountability Act
PHI: protected health information
REACHnet: Research Action for Health Networks
REDCap: Research Electronic Data Capture
